# A precision translation stage for reproducing measured target volume motions

**DOI:** 10.1120/jacmp.v8i3.2221

**Published:** 2007-08-08

**Authors:** Dale W. Litzenberg, Scott W. Hadley, Kwok L. Lam, James M. Balter

**Affiliations:** ^1^ Department of Radiation Oncology University of Michigan Health System Ann Arbor Michigan U.S.A.

**Keywords:** Intrafraction motion, 4D imaging, respiratory motion, patient motion simulation

## Abstract

The development of four‐dimensional (4D) imaging, treatment planning, and treatment delivery methods for radiation therapy requires a quality assurance device that can reproduce clinical motions. Here, we present a high‐precision translation stage for testing and validating 4D techniques. These techniques may require spatial resolutions of 1 mm and temporal resolutions of 2 – 30 Hz for computed tomography imaging, electromagnetic tracking, and fluoroscopic imaging. A one‐dimensional programmable translation stage capable of reproducing idealized and measured anatomic motions common to the thorax and providing support for phantoms weighing up to 27 kg was designed and built to meet the foregoing spatial and temporal resolution requirements. The stage consists of a polycarbonate base and table, driven by an ac servo motor with encoder feedback by means of a belt‐coupled precision screw. Complex motions are made possible by a programmable motion controller that can run multiple independent control and monitoring programs concurrently on independent motion axes. Programmable input and output ports allow motion to be synchronized with beam delivery and other imaging and treatment delivery devices to within 2.0 ms. Average deviations from the programmed positions are typically 0.2 mm or less, and the average maximum positional errors are typically 0.5 mm for an indefinite number of parameterized breathing motion cycles during reproduction of measured target volume motions for several minutes.

PACS number: 87.66.Xa

## I. INTRODUCTION

As in‐room image guidance and target volume tracking technology advances, errors of setup and interfraction target volume position are greatly reduced, and explicit knowledge of intrafraction motion becomes more important in both treatment planning and delivery. Treatment planning methods are now beginning to use four‐dimensional (4D) computed tomography (CT) and 4D cone‐beam CT for improved target delineation, to convolve patient‐specific target motion with static dose distributions, and to specify delivery parameters for target volume tracking and beam gating. The development, testing, and validation of these imaging, planning, and delivery technologies requires dosimetric measurements in phantoms that move with similar patient‐specific motions. Likewise, it is necessary to know the position of a phantom with an uncertainty that is comparable to the spatial resolution of the imaging system and at time intervals that are comparable to the imaging frequency. Computed tomography scans for treatment planning may have slice thicknesses as small as 1 mm and may acquire an image in less than 0.5 s.

Several translation stages have been reported in the literature.^(^ ^1^ ^–^ ^4^ ^)^ One high‐precision stage reported for use with tomotherapy employed a rotary table on a translation stage.^(1)^ Others have used a rotary drive motor to produce sinusoidal motion in a translation stage holding detectors or phantoms,^(^ ^2^ ^,^ ^3^ ^)^ and another used a programmable stepper motor coupled to a translation stage with a lead screw to simulate the irregular breathing motion observed in patients.^(4)^

Those stages provide a wide range of capabilities for simulating motion in phantoms of varying mass, with varying degrees of accuracy. Rotary induction motors may provide a reasonably accurate cyclical motion once at constant speed, but they are limited to sinusoidal motions, which are not wholly representative of observed clinical anatomic motion. Stepper motors are capable of high‐resolution incremental motion, but they are susceptible to “slippage” between steps at higher speeds, where lower motor torque is insufficient to produce the desired change in momentum of the coupled inertial load. The slippage may be reduced by reducing the weight of the phantom or by reducing the instantaneous velocity and acceleration of the motion profile to achievable values. A closed‐loop positional feedback mechanism may also be implemented to account for slippage and to help ensure that the desired location is eventually reached, although at a potentially different time than that desired.

In the present work, we report on the development of a programmable translation stage capable of reproducing idealized and measured motions with a spatial resolution of 0.5 mm or better at any given time, within design limits. In addition, motion may be synchronized with image acquisition or treatment delivery to within 2 ms, for applications such as verifying dose reconstructions from clinically measured data^(5)^ and driving deformable phantoms for verification of dynamic imaging and dose delivery modeling.^(6)^

## II. MATERIALS AND METHODS

### A. Stage design and control

**Figure 1:**
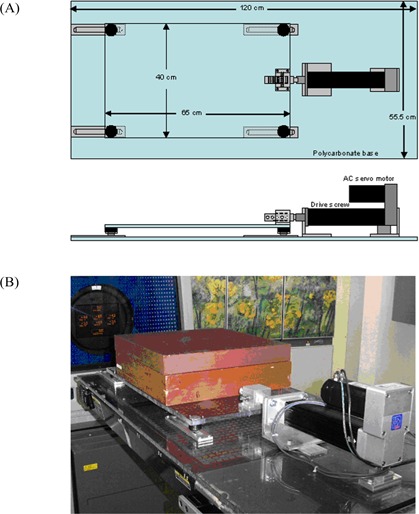
Translation stage. (A) Schematic and (B) picture of the programmable translation stage, designed to reproduce motion profiles with less than 0.5 mm positional error for amplitudes up to 60 mm, velocities up to 80 mm/s, and accelerations up to $100\;\text{mm}/s^{2}$ with a phantom up to 27 kg. AC=alternating current.

The stage, shown in Fig. 1, consists of a polycarbonate base and translation platform, 12.7 mm thick. Precision‐ground steel casters are mounted to the platform and ride in milled aluminum tracks. The inner distance between the forward and aft tracks is 460 mm, which allows an ample region to be CT‐scanned without introducing significant artifacts. Driving the stage is an electric cylinder (Model EC2: Industrial Devices Corporation, Novato, CA) with an Acme ball screw (length: 100 mm; diameter: 15 mm; pitch: 2 mm), which is belt‐driven through a 3:2 turn‐ratio gear coupling by an AC servo motor (Model AKM23D: Danaher Corporation, Washington, DC) with position‐encoded feedback. The servo motor is controlled by a servo driver (Model S200: Danaher Corporation), which is slaved to a programmable motion controller (Model MC202: Trio Motion Technology, Pittsburgh, PA) with position feedback. The default factory‐supplied motor tuning parameters are used.

Serial communication between the motion controller and a host computer allows for motion programs to be downloaded to the controller. In the programming environment, the user can use a modified BASIC programming language to create complex motion control programs, with user input and communication with other devices through input/output (I/O) ports, and to call predefined routines that coordinate and synchronize complex motions. Analytic and arbitrary motion profiles can thereby be downloaded to the controller's memory (limited to 8000 positions for this particular model), and the profiles can then be executed within a user‐specified time period. Profiles described by an analytic function are calculated at uniform time intervals; arbitrary or measured profiles are sampled at uniform time intervals. The servo control system receives positional feedback at 1‐ms intervals and attempts to minimize the difference between the desired and actual profiles, given the servo tuning parameters (factory defaults were used) and the inertial properties of the system. Motion programs can loop over a specified range of positions an arbitrary number of times (≥ 1). The system was designed to reproduce analytic and arbitrary motion profiles with ≤0.5‐mm positional error for amplitudes up to 60 mm, velocities up to 80 mm/s, and accelerations up to $100\;\text{mm}/s^{2}$ with a phantom of up to 27 kg.

**Figure 2:**
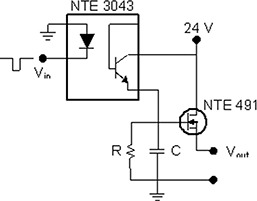
Trigger circuit. Diagram of the circuit used to trigger motion based on arrival of the first beam pulse. The input pulse is taken from the bremsstrahlung target of the accelerator and supplies a 24‐V logic signal to an input/output port on the programmable motion controller.

In addition, the programmable I/O ports are used as a method of synchronizing the beginning of motion with image acquisition or with treatment beam delivery, such as that from a Varian 2100 EX (Varian Medical Systems, Palo Alto, CA), as in the present case. Synchronization is accomplished simply by waiting for an appropriate logic input signal (in this case, nominally 24 volts) before initiating motion in the control program. For treatment delivery synchronization, the logic pulse is provided by the simple circuit shown in Fig. 2, which consists of an optoisolator, resistor (R=100 MΩ), capacitor (C=400 pF), and a field‐effect transistor. Here, the negative signal from the bremsstrahlung target (which is roughly a square pulse of −1.5 V, 3 ms for 6‐MV photons) is input into the optoisolator, which is used to charge the capacitor and trigger the field‐effect transistor, which applies approximately 24 V to the input channel on the motion controller. The resistor is chosen so that the time constant, $\underset{˙}{t}=\text{RC}$, is long compared to the time between input pulses.

### B. Motion profile preparation

The system was designed to reproduce analytic and arbitrary profiles of superior–inferior motion for amplitudes up to 60 mm, velocities up to 80 mm/s, and accelerations up to $100\;\text{mm}/s^{2}$. Motion profiles (positions only, approximately 50 per second of motion) generated from analytic functions that meet these requirements may be downloaded directly to the motion controller in the appropriate ASCII file format. Motion profiles obtained from real patients include measurement noise that may need to be smoothed for better reproducibility. Smoothing may be performed globally or locally until the speed and acceleration specifications of the stage are satisfied. For example, test profiles measured with an electromagnetic tracking system (Calypso Medical Technologies, Seattle, WA) contain readout noise of <0.5 mm at 10 Hz.^(^ ^7^ ^,^ ^8^ ^)^ Profiles of this kind have been reproduced without smoothing with good results, but smoothing is typically applied to reduce unnecessary stress on the motor and drive belt. A global sliding‐average smoothing (“boxcar smoothing”) is typically applied ten times with a small number of elements (n=5), closely preserving the local amplitude and slope characteristics of the underlying motion data. It is important that the first few seconds of the motion profile meet the velocity and acceleration criteria defined earlier so that large instantaneous initial velocities and accelerations are avoided. Otherwise, positional errors of several millimeters may result in the first few seconds until the velocity and acceleration of the motion profile are within the specified criteria.

### C. Motion profile reproduction tests

To test the capabilities and accuracy of the system, idealized and measured motion profiles were downloaded to the motion controller, were executed, and were measured using the Varian Respiratory Position Management (RPM) system (Varian Medical Systems, Palo Alto, CA). An analytic breathing function of the form $x(t)=A\cos^{4}\;(\pi t/T)$, was tested for amplitudes, *A*, ranging from 5 mm to 20 mm, and periods, *T*, of 2, 4, and 6 s, over a 45‐s measurement period. This commonly used analytic function, based on the work of Lujan and colleagues,^(9)^ was chosen because it can easily be used to test the velocity and acceleration characteristics of the stage with values of clinical interest. Four measured profiles with excursions of more than 10 mm, lasting approximately 60 s were reproduced and measured for several minutes. Phantom weights between 0 kg and 27.3 kg (60 lb.) were used.

### D. Motion–beam synchronization test

The delay between the start of the beam and the beginning of motion was measured by examining the time delay between the arrival of the input signal and the time for an immediately switched output channel on the motion controller to reach full voltage. It was assumed that full voltage could be applied to the internal circuit that initiates motion with similar timing characteristics. This delay was simply measured 10 times with a digital storage oscilloscope in single‐shot trigger mode.

## III. RESULTS AND DISCUSSION

### A. Motion profiles generated from analytical functions

**Figure 3:**
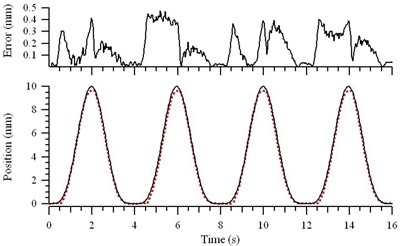
Analytical motion reproduction. Four cycles of an idealized typical breathing motion (solid black line), $x(t)=10\cos^{4}(\pi t/4)$ mm, are shown with the motion reproduced by the translation stage as measured with the Respiratory Position Management system [Varian Medical Systems, Palo Alto, CA (dotted red line)]. The residual position errors are shown at the top.

**Figure 4:**
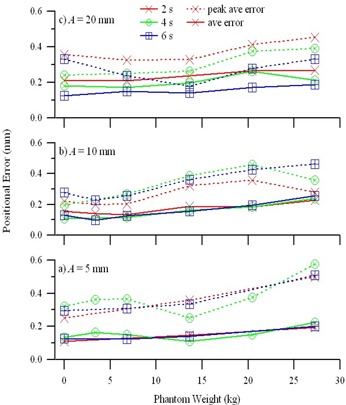
Accuracy results. The average error (solid lines) and “average peak error” (dashed lines) versus phantom weight, over the entire 45‐s measurement period for idealized breathing motion profiles. Errors for motion profiles with periods of 2 s (red crosses), 4 s (green circles), and 6 s (blue squares) are shown for amplitudes of (a) A=5 mm, (b) A=10 mm, and (c) A=20 mm.

Fig. 3 shows an example of four periods of the analytic motion profile $x(t)=A\cos^{4}\;(\pi t/T)$ mm that was downloaded to the motion controller; the motion profile that was measured with an optical tracking system (RPM); and the absolute position error. As expected, the errors were largest during and after periods of the largest acceleration. The functional form was generally well reproduced, but the measured amplitude at time t=T/2, was consistently less than the programmed motion. Fig. 4 shows the average error over the entire measurement period, as well as the “average peak error,” or the average of the errors at times $t_{n}=(n+1/2)T<T_{\text{Meas}}$ during the measurement period, where *n* is an integer greater than or equal to zero. For each amplitude, A=5 mm, 10 mm, and 20 mm, the average and “average peak error” is shown for periods T=2 s, 4 s, and 6 s, and for several phantom weights ranging from 0 kg to 27.3 kg. Although the position errors increase with increased phantom weight, the average errors are typically below 0.25 mm (range: 0.10 – 0.27 mm), and the “average peak error” is typically less than 0.5 mm (range: 0.18 – 0.58 mm). For a typical water‐equivalent phantom measuring 300×300×150 mm, which weighs approximately 13.5 kg, the average error is 0.16 mm, and the average peak error is 0.39 mm.

### B. Motion profiles generated from measured data

**Figure 5:**
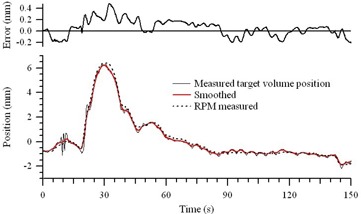
Measured motion reproduction. The solid line shows the raw position versus time data of a supine prostate target volume as measured with the Calypso 4D Localization System (Calypso Medical Technologies, Seattle, WA). The smoothed profile that was downloaded to the motion controller is shown in red, and the reproduce motion profile, measured with the Respiratory Position Management system (Varian Medical Systems, Palo Alto, CA), is shown by the dashed line. The residual position errors are shown at the top.

Fig. 5 shows the raw motion data from measurement of prostate motion in a patient under an internal review board–approved protocol and using the Calypso electromagnetic tracking system. The data was smoothed with 50 passes of a 5‐element sliding average (as shown) and downloaded to the translation stage; the reproduced motion was then measured using the RPM system. The residual position errors between the smoothed and reproduced motion profiles are also shown. The form of the original motion profile is reproduced with a root‐mean‐square (RMS) residual error of 0.15 mm and a maximum residual error of 0.49 mm with a 27.3 kg phantom. From the form of the residual errors, the features of approximately 0.2 mm over a few seconds are seen not to be well reproduced with the motor tuning parameters used to obtain the results reported here. Four motion profiles (including the one shown in Fig. 5) where each measured 5 times. The average RMS error was 0.18 mm (range: 0.14 – 0.30 mm), and the average maximum error was 0.52 mm (range: 0.38 – 1.05 mm).

The system was designed to reproduce motion profiles for amplitudes up to 60 mm, velocities up to 80 mm/s, and accelerations up to $100\;\text{mm}/s^{2}$. These values represent the most challenging that are likely to be encountered in clinical practice. In addition, the system was overdesigned so that these criteria would not reach the physical capability limits of the system. The clinically relevant profiles tested here were reproduced within the expected uncertainties, and the limits of the system were not thoroughly explored.

### C. Motion–beam synchronization results

The signal from the bremsstrahlung target was used by the circuit in Fig. 2 to create a 24‐V logic pulse that was used as a trigger on an I/O port to initiate motion in the translation stage. The delay between the arrival of the signal from the target and the time at which the voltage on another output port reached 90% of its final voltage (30 μs rise‐time to 90% final voltage) was measured 10 times and was assumed to represent the time to initiate motion. The average time delay was 2.0 ms (range: 1.3 – 3.9 ms) with a standard deviation of 0.7 ms.

Other features of the system that may also be explored in the future include the use of multiple independent processes that can run concurrently on the motion controller. These capabilities will allow beam gating through programmable I/O ports and position recording at the time of each beam pulse.

## IV. CONCLUSIONS

The programmable translation stage described in this work is capable of reproducing idealized and measured anatomic motions commonly observed in the thorax with roughly 0.2 mm average error and typical maximum errors of 0.5 mm for phantoms weighing up to approximately 27 kg. In addition, the motion can be synchronized to the beginning of the treatment beam with an average delay of 2.0 ms. Consequently, this system could be used to test, develop, and validate treatment planning and delivery technologies for moving target volumes where spatial resolutions of 1 mm are becoming common. In addition, because of its temporal synchronization capabilities, the system could be used to validate new imaging technologies such as 4D CT, real‐time fluoroscopic imaging, and electromagnetic target volume tracking.

## ACKNOWLEDGMENTS

We acknowledge the contributions of Yin Ren in writing motion control software, and in collecting and analyzing preliminary data on the capabilities of the system. This work was supported in part by National Institutes of Health Grant P01‐CA59827.
